# Exploring the Structural and Functional Consequences of Deleterious Missense Nonsynonymous SNPs in the *EPOR* Gene: A Computational Approach

**DOI:** 10.3390/jpm14111111

**Published:** 2024-11-20

**Authors:** Elshazali Widaa Ali, Khalid Mohamed Adam, Mohamed E. Elangeeb, Elsadig Mohamed Ahmed, Hytham Ahmed Abuagla, Abubakr Ali Elamin MohamedAhmed, Ali M. Edris, Elmoiz Idris Eltieb, Hiba Mahgoub Ali Osman, Ebtehal Saleh Idris

**Affiliations:** Department of Medical Laboratory Sciences, College of Applied Medical Sciences, University of Bisha, P.O. Box 551, Bisha 67714, Saudi Arabia; kmabdalla@ub.edu.sa (K.M.A.); melnageeb@ub.ed.sa (M.E.E.); emfadlalla@ub.edu.sa (E.M.A.); aadlah@ub.edu.sa (H.A.A.); aaelamin@ub.edu.sa (A.A.E.M.); aedris@ub.edu.sa (A.M.E.); elmoizie@ub.edu.sa (E.I.E.); hmosman@ub.edu.sa (H.M.A.O.); ebtihal@ub.edu.sa (E.S.I.)

**Keywords:** erythropoietin receptor, *EPOR* gene, nonsynonymous single nucleotide polymorphisms

## Abstract

Background: Mutations in the *EPOR* gene can disrupt its normal signaling pathways, leading to hematological disorders such as polycythemia vera and other myeloproliferative diseases. Methodology: In this study, a range of bioinformatics tools, including SIFT, PolyPhen-2, SNAP2, SNPs & Go, PhD-SNP, I-Mutant2.0, MuPro, MutPred, ConSurf, HOPE, and Interpro were used to assess the deleterious effects of missense nonsynonymous single nucleotide polymorphisms (nsSNPs) on protein structure and function. Furthermore, molecular dynamics simulations (MDS) were conducted to assess the structural deviations of the identified mutant variants in comparison to the wild type. Results: The results identified two nsSNPs, R223P and G302S, as deleterious, significantly affecting protein structure and function. Both substitutions occur in functionally conserved regions and are predicted to be pathogenic, associated with altered molecular mechanisms. The MDSs indicated that while the wild-type EPOR maintained optimal stability, the G302S and R223P variants exhibited substantial deviations, adversely affecting overall protein stability and compactness. Conclusions: The computational analysis of missense nsSNPs in the *EPOR* gene identified two missense SNPs, R223P and G302S, as deleterious, occurring at highly conserved regions, and having substantial effects on erythropoietin receptor (EPO-R) protein structure and function, suggesting their potential pathogenic consequences.

## 1. Introduction

Human erythropoietin (EPO) is a peptide hormone produced in the fetal liver during early development and by the kidneys in adults. As a critical hematopoietic growth factor (HGF), EPO regulates erythropoiesis in the bone marrow, driving the production of around 200 billion red blood cells (RBCs) daily [1]. Upon binding to its receptor, EPO activates the Janus kinase/signal transducer and activator of the transcription (JAK/STAT) signaling pathway. This pathway promotes the proliferation of erythroid progenitor cells and protects them from apoptosis [2]. The activation of STAT5 is critically dependent on specific tyrosine residues within the cytosolic domain of the erythropoietin receptor (EPO-R). Following the binding of EPO to its receptor, a receptor conformational change occurs, leading to the activation of JAK2. This results in the phosphorylation of multiple tyrosine residues in the cytoplasmic domain of EPO-R, which are essential for downstream signaling pathways, including the activation of STAT5 [3,4,5].

Beyond its role in erythropoiesis, EPO also exhibits pleiotropic effects across various tissues and organs. Studies have identified EPO and EPO-R expression in the brain, as well as in the nervous and respiratory systems [6,7,8]. EPO has gained recognition for its neuroprotective properties, with studies showing its ability to enhance outcomes following traumatic brain injury and protect retinal neurons from ischemia-reperfusion injury. It has also been explored for its potential cardioprotective effects in patients with myocardial infarction [9] and it has been discovered to regulate energy metabolism [10].

EPO-R is a member of the cytokine receptor superfamily, which also includes receptors for other hematopoietic growth factors such as growth hormone, prolactin, granulocyte colony-stimulating factor (G-CSF), granulocyte-macrophage colony-stimulating factor (GM-CSF), thrombopoietin, oncostatin M, and various interleukins. Receptors in this family share common structural characteristics: an extracellular ligand-binding domain with two pairs of conserved cysteine residues and a WSXWS motif near the transmembrane domain, a single transmembrane domain, and an intracellular domain that lacks catalytic activity [11]. EPO-R mRNA, binding sites, and associated signaling pathways have been identified in various non-hematopoietic tissues, including the heart, blood vessels, kidneys, liver, gastrointestinal tract, pancreatic islets, testis, female reproductive system, and placenta [12]. The involvement of EPO-R in human diseases, particularly in conditions such as polycythemia vera and hereditary polycythemia, has been extensively studied. Structurally abnormal *EPOR* genes have been identified in patients with primary familial and congenital polycythemia, suggesting a possible connection between *EPOR* mutations and erythrocytosis [13].

In silico analysis of single nucleotide polymorphisms (SNPs) is a powerful approach for investigating genetic variations linked to clinical conditions. Computational tools and algorithms allow the identification and analysis of candidate SNPs, providing valuable insights into their potential effects on human health and disease [14,15,16,17,18]. Moreover, in silico analysis of gene variants is of utmost importance in pharmacogenomics, enabling the identification of high-risk variants influencing drug responses. This approach supports the development of personalized medicine and aids in discovering novel therapeutic and diagnostic markers [14].

This study aimed to analyze missense nonsynonymous SNPs in the *EPOR* gene and evaluate their deleterious effects on protein structure and function, as well as their potential disease associations, using computational tools.

## 2. Materials and Methods

### 2.1. Work Plan

**Figure 1:**
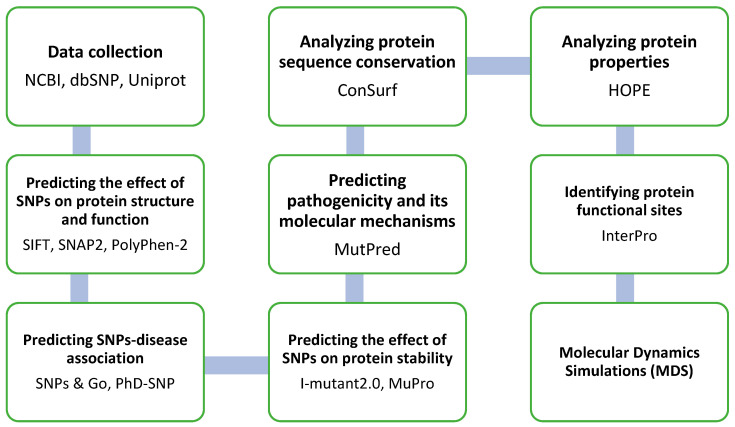
Flowchart outlining the identification and categorization of nonsynonymous single nucleotide polymorphisms (nsSNPs) in the *EPOR* gene, with each step indicating the tools used. If an nsSNP is classified as deleterious by a particular tool at any step, it progresses to the next tool or step for further analysis. In the last two steps, tools were utilized to examine and visualize structural alterations.

Several computational tools and algorithms were employed in this study to explore the impact of missense nsSNPs in the *EPOR* gene on the structure and function of the EPO-R protein. Through a systematic approach, we explore the vast landscape of missense nsSNPs within this gene, predicting their potential consequences at molecular and phenotypic levels and their associations with diseases (Figure 1).

### 2.2. Data Collection

Data pertaining to the human *EPOR* gene (ID: 2057) and its nucleotide (NG_021395) and amino acid (NP_000112.1) sequences were sourced from NCBI (https://www.ncbi.nlm.nih.gov/) (accessed on 11 November 2023). The missense nsSNPs located within the EPOR gene were obtained from the dbSNP database (http://www.ncbi.nlm.nih.gov/SNP/) (accessed on 11 November 2023). The protein sequence for EPO-R (P19235) in FASTA format was obtained from the UniProt database (http://www.uniprot.org/uniprot/) (accessed on 11 November 2023). 

### 2.3. Identification of the Deleterious nsSNPs in the EPOR Gene

To assess the impact of missense nsSNPs on EPO-R protein structure and function, we employed several bioinformatics tools, including SIFT, PolyPhen-2, SNAP2, SNPs & Go, PhD-SNP, I-Mutant2.0, MuPro, MutPred, ConSurf, HOPE, and InterPro, to analyze all reported missense nsSNPs in the *EPOR* gene. The SNPs classified as deleterious by these tools were further processed for molecular dynamics simulations (MDS). Furthermore, PyMol was used to display the three-dimensional (3D) structural changes caused by deleterious SNPs.

### 2.4. Predicting the Effect of SNPs on EPO-R Protein Structure and Function

Three computational tools, FIFT, SNAP2, and PolyPhen-2, were used to predict the effect of nsSNPs on protein structure and function.

The SIFT (Sorting Intolerant from Tolerant) tool (https://sift.bii.a-star.edu.sg/) (accessed on 15 November 2023) predicts the impact of amino acid substitutions on protein function by analyzing sequence conservation and physicochemical properties. The rsIDs obtained from the dbSNP database were used as input queries, and the substitutions with a SIFT score < 0.05 were categorized as deleterious [19].

SNAP2 (Screening for Non-acceptable Polymorphisms) (https://www.rostlab.org/services/SNAP2) (accessed on 17 November 2023) differentiates between damaging and neutral variants by analyzing sequence and variant properties, providing a score to indicate whether a variant is likely to be deleterious. [20,21].

PolyPhen-2 (Polymorphism Phenotyping v2) (http://genetics.bwh.harvard.edu/pph2/) (accessed on 28 November 2023) combines sequence-based and structural data to classify variants as “benign,” “possibly damaging,” or “probably damaging.” It uses a Bayes posterior probability, with scores ranging from 0.0 to 1.0, to evaluate the likelihood of a harmful substitution [21,22,23].

### 2.5. Prediction of SNP-Disease Associations

SNPs & GO (Screening for Non-Acceptable Polymorphisms) and PhD-SNP (Predictor of Human Deleterious Single Nucleotide Polymorphisms) tools explored SNPs-disease association.

The SNPs & GO (http://snps-and-go.biocomp.unibo.it/snps-and-go/) (accessed on 4 December 2023) tool combines protein sequence information with functional annotations from Gene Ontology (GO) terms to predict the deleterious effects of human protein variants. By integrating sequence data with GO-based functional insights, it improves the accuracy of determining whether a mutation is disease-related, helping to assess the potential impact of genetic variations on protein function. A reliability index (R1), ranging from 0 to 10, measures the confidence or reliability of the prediction made by the tool [24,25,26].

PhD-SNP (https://snps.biofold.org/phd-snp/phd-snp.html) (accessed on 11 December 2023) uses a support vector machine (SVM) algorithm to predict the functional impact of protein mutations, particularly single amino acid substitutions. It assesses various features, including sequence conservation, physicochemical properties, and functional annotations, to classify SNPs as disease-associated or neutral. A score above 0.5 suggests that the mutation may be pathogenic [27].

### 2.6. Predicting the Effect of SNPs on Protein Stability

I-Mutant 2.0 and MuPro tools were used to assess the impact of amino acid changes on protein stability.

I-Mutant2.0 (https://folding.biofold.org/i-mutant/i-mutant2.0.html) (accessed on 15 December 2023) employs the SVM algorithm to predict how missense nsSNPs affect protein stability. The tool requires input data including the protein sequence, the specific mutated residues, and their positions. It generates a reliability index (RI) ranging from 0 to 10, with 10 indicating the highest level of prediction reliability [28].

MuPro (http://mupro.proteomics.ics.uci.edu/) (accessed on 23 December 2023) is an advanced online tool for predicting how single-site amino acid mutations affect protein stability. It uses machine learning techniques, including SVM and neural networks, to assess the impact of SNPs [20,29,30]. The tool accepts protein sequences in FASTA format, enabling users to predict stability changes without needing the protein’s tertiary structure [31]. MuPro’s main output is the change in free energy (ΔΔG) caused by the mutation, which is calculated using SVM trained on a large mutation dataset and has an accuracy above 84% via 20-fold cross-validation, indicating whether the mutation stabilizes or destabilizes the protein. A ΔΔG value below zero suggests reduced stability, while a value above zero indicates increased stability [32,33].

### 2.7. Predicting Pathogenicity and Its Molecular Mechanism

MutPred (http://mutpred.mutdb.org) (accessed on 28 December 2023) was utilized to classify SNPs as pathogenic or benign. It is a sophisticated computational tool developed to predict the impact of amino acid variants on protein function and stability. It improves upon existing methods by prioritizing pathogenic amino acid substitutions, proposing potential disease-causing molecular mechanisms, and offering interpretable pathogenicity score distributions for individual genomes. Using a random forest-based classification approach, MutPred2.0 evaluates 14 different structural and functional protein properties—such as helical propensity and the loss of phosphorylation sites—combined with evolutionary conservation data to assess the likelihood that an amino acid variant will have a phenotypic effect [34].

### 2.8. Assessing the Conservation of Amino Acid Positions 

The ConSurf server (https://consurf.tau.ac.il/) (accessed on 29 December 2023) was used to analyze protein sequence conservation. It is specifically designed for assessing the evolutionary conservation of amino acid positions within a protein. The rate of evolutionary change at a particular amino acid or nucleic acid location is directly linked to the structural and functional significance of that amino acid. To carry out this computation, ConSurf uses either the maximum likelihood (ML) approach or the empirical Bayesian method. The results are presented in a color-coded format that highlights conservation scores, categorized into three groups: variable, average, and highly conserved regions. It generates scores on a scale from 1 to 9. The protein sequence in FASTA format was used as an input query [35].

### 2.9. Analyzing Protein Properties

HOPE tool (https://www3.cmbi.umcn.nl/hope/input/) (accessed on 30 December 2023) was applied to analyze protein properties. It automates the assessment of how single nucleotide alterations influence both the structural and functional attributes of proteins, drawing from data found in the UniProt database. This tool offers a comprehensive portrayal of the mutation’s consequences, generating detailed reports that encompass written descriptions, visual graphics, and interactive visualizations [36].

### 2.10. Identifying the Protein Functional Sites

InterPro (https://www.ebi.ac.uk/interpro/) (accessed on 25 October 2024) was used to predict the functional site of the SNPs. It is a major resource for protein sequence analysis that incorporates multiple databases, including Pfam, SMART, ProDom, and others, to provide thorough annotations of protein families, domains, and functional sites. The tool employs predictive models, or signatures, derived from these databases, allowing the classification of proteins based on their sequence data and predicting their functional roles [37,38].

### 2.11. Conducting Molecular Dynamics Simulations

Molecular dynamics simulations (MDS) were performed using GROMACS version 2020.6 on a Google Colab Pro notebook to investigate structural changes over time in both wild-type and mutant structures. For initial calculations, the GROMACS-OPLS-AA force field was employed. The structures were placed in a cubic box, partially filled with water molecules up to a 1 nm margin. The system was neutralized by adding 10 sodium ions (Na+) using the GROMACS genion tool.

Energy minimization was conducted using the steepest descent algorithm with an energy step size of 0.01 and a maximum of 50,000 iterations. To stabilize the system, a 1 bar Parrinello–Rahman pressure coupling (pcouple) and a 300 K Berendsen temperature coupling (tcouple) were applied, with coupling constants set at 2.0 ps for pressure and 0.1 ps for temperature. The partial mesh Ewald (PME) method was utilized for calculating electrostatic interactions, with short-range cutoffs of 1.0 nm for both van der Waals (rvdw) and electrostatic (rcoulomb) interactions. The neighbor list (nstlist) was updated every 10 ps, and all bond constraints, including those involving heavy atoms and hydrogen bonds, were maintained using the LINCS algorithm with a time step of 0.002 ps. An isothermal compressibility of 4.5 × 10^−5^ was used.

The system was equilibrated in both NVT (constant number of particles, volume, and temperature) and NPT (constant number of particles, pressure, and temperature) ensembles for 100 ps, maintaining the pressure at 1 bar and temperature at 300 K using the Parrinello–Rahman and Berendsen methods, respectively. Subsequently, 10 ns molecular dynamics simulations were conducted for both wild-type and mutant structures, with trajectories recorded every 1 ps.

The GROMACS and XMGRACE programs were used to calculate and plot the root-mean-square deviation (RMSD), root-mean-square fluctuation (RMSF), radius of gyration (Rg), number of hydrogen bonds, and solvent-accessible surface area (SASA). These analyses facilitated a comparative assessment of structural deviations between wild-type and mutant structures [39].

### 2.12. Displaying 3D Structural Change Using PyMol Software

A 3D simulation of the two variants predicted as deleterious by various bioinformatic tools and MDS was conducted to visualize structural changes using PyMol software, version 2.0, which is a molecular visualization tool extensively used in structural biology research. It enables the creation of detailed 3D representations of biomolecules, including proteins [20,40].

## 3. Results

### 3.1. Effect of nsSNPs on Protein Structure and Function

The analysis of nsSNPs in the *EPOR* gene revealed valuable insights into its structural and functional stability. Of the 420 missense nsSNPs examined, 77 were identified as deleterious by the SIFT, PolyPhen-2, and SNAP2 tools. SIFT classified these SNPs as “Deleterious,” PolyPhen-2 marked them as “Probably damaging,” and SNAP2 indicated they would have an “Effect.” These findings suggest these SNPs may negatively affect the protein’s function (Table 1).

### 3.2. Predicting SNPs-Disease Association

Out of the 77 nsSNPs classified as deleterious, 47 were predicted as disease-associated using the SNPs & Go and PhD-SNP tools (Table 2).

### 3.3. Analyzing the Impact of SNPs on Protein Stability

The use of the I-Mutant2.0 and MuPro tools provided insightful predictions regarding the impact of the nsSNPs categorized as disease-associated on protein stability. Out of the 47 mutations analyzed, 34 were predicted to decrease protein stability (Table 3).

### 3.4. Predicting the Molecular Mechanism of Pathogenicity

The MutPred tool was used to predict the potential molecular mechanisms affected by specific amino acid changes caused by nsSNPs. Seventeen SNPs were predicted to disturb the molecular mechanisms. Key mechanisms affected include gain of strand, gain of ADP-ribosylation, altered stability, altered transmembrane protein, loss of disulfide linkage, altered ordered interface, gain of O-linked glycosylation, loss of pyrrolidone carboxylic acid, gain of loop, and altered disordered interface (Table 4).

### 3.5. Analyzing Protein Sequence Conservation

**Figure 2:**
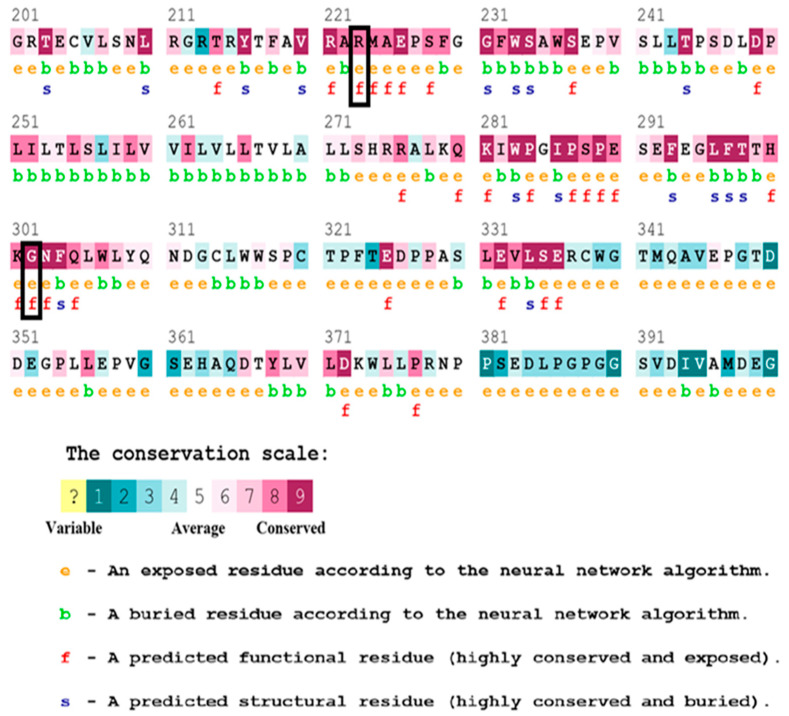
Outcomes of the conservation analysis using the ConSurf tool display sequence conservation using a color gradient. In this scheme, sky blue represents variable residues, while dark purple indicates highly conserved residues. Functional residues are marked with “f”, while structural residues are marked with “s”. Buried (b) and exposed (e) residues are also distinguished, showing their potential interactions within the protein or with external molecules. The two mutant variants (in the boxes) are situated in exposed-functionally conserved positions.

The conservation of amino acid residues that are substituted in various nsSNPs was analyzed using the ConSurf tool. The analysis revealed that of the 17 SNPs predicted to disturb the molecular mechanism, two residues, R223P and G302S, are predicted to be functional and exposed residues with conservation scores of 8 and 9, respectively, highlighting their significant role in the protein’s function. Mutations in such residues are likely to have deleterious effects due to their involvement in critical processes. (Figure 2).

### 3.6. Analysis of Protein Properties

The analysis of protein properties was carried out using HOPE tool. The rs991881188 changes the amino acid at position 223 from arginine (R) to proline (P). Arginine, which is positively charged and highly flexible, is replaced by proline, a smaller and more hydrophobic residue. This substitution disrupts critical ionic interactions and hydrogen bonds, specifically affecting interactions with glycine at position 231 and forming salt bridges with glutamic acids at positions 181, 197, and 226. The change in charge from positive to neutral also disturbs the interactions, impacting the protein’s stability. The MetaRNN score of 0.8490628 indicates a significant likelihood that this mutation has a harmful impact.

The rs1321784132 changes the amino acid at position 302 from glycine (G) to serine (S). Glycine, a small amino acid, is replaced by the larger amino acid, serine, which can lead to significant structural alterations. The two amino acids are neutral in terms of charge, but the substitution can cause the loss of interactions with other molecules due to the difference in size and placement. Serine is incorrectly positioned to make the same hydrogen bond as glycine, potentially disrupting local structural stability. This substitution is near a highly conserved region, indicating its importance in protein function. The structural impact is considerable, as the substitution can force the local backbone into an incorrect conformation, abolishing its function and disturbing the local structure. The MetaRNN score of 0.8904699 suggests a high probability that this substitution is deleterious.

### 3.7. Predicting the Protein Functional Sites

The analysis of the protein functional sites using InterPro revealed that the R223P polymorphism is located in the fibronectin type III domain (FN3), which shows functional and structural modularity, with key interaction sites mapped to short amino acid sequences like Arg-Gly-Asp (RGD). This RGD sequence is critical in binding integrins, and RGD-containing peptides can influence cell adhesion events. The other SNP, G302S, is located within the cytoplasmic domain of the EPO-R, which is thought to be important for interaction with common signal transducers or protein tyrosine kinases.

### 3.8. Molecular Dynamics Simulations of Wild Type and Mutant Variants

#### 3.8.1. Root-Mean-Square Deviation (RMSD)

**Figure 3:**
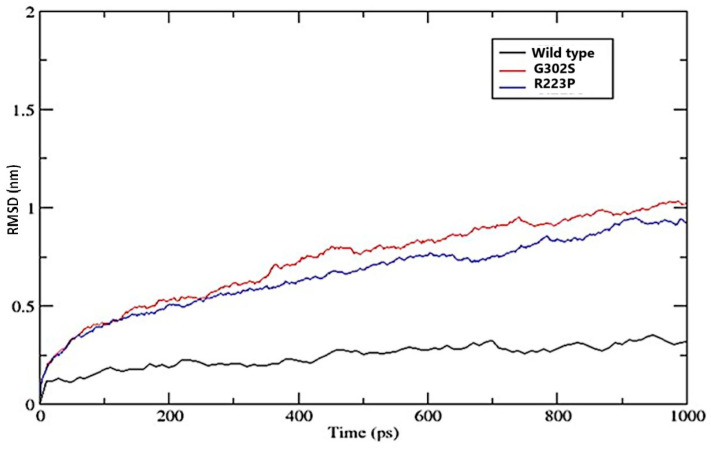
The Root-Mean-Square Deviation (RMSD) plot compares the structural stability of the wild-type protein with two mutant variants, G302S and R223P. The y-axis represents the RMSD in nanometers (nm), while the x-axis shows time in picoseconds (ps). The wild type is represented in black, with the G302S and R223P mutants represented in red and blue, respectively.

The wild-type EPOR variant displayed minimal deviation from its initial structure, starting just above 0 nm and gradually increasing to approximately 0.3 nm. This suggests a stable conformation throughout the simulation period. In contrast, the G320S variant began around 0.2 nm and steadily raised to greater than 1.0 nm, indicating a significant deviation from its initial structure and suggesting less structural stability. The R223P variant started close to the wild type but slowly increased and diverged, reaching about 1 nm. While it exhibited greater stability than the G320S variant, it is less stable than the wild type (Figure 3).

#### 3.8.2. Root-Mean-Square Fluctuation (RMSF)

**Figure 4:**
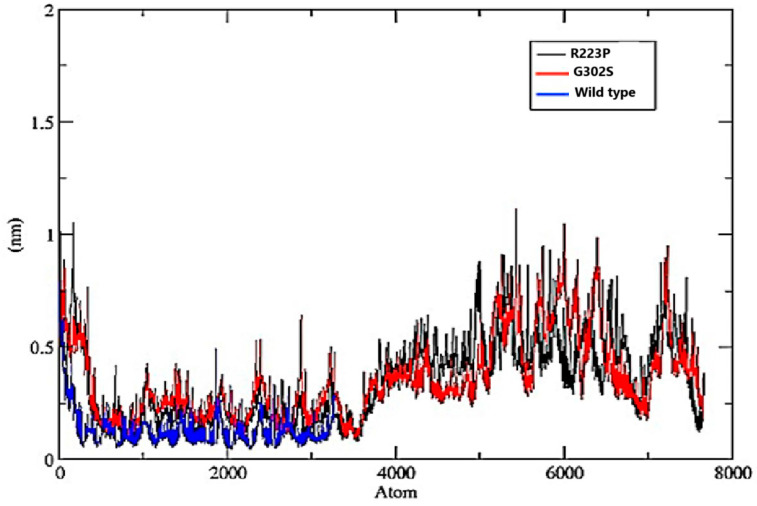
The Root-Mean-Square Fluctuation (RMSF) plot for three different protein variants, R223S (black), G302S (red), and wild type (blue). The y-axis represents the fluctuation in nanometers (nm), while the x-axis represents the atomic positions in the protein chain.

The wild-type variant exhibited consistently lower fluctuations across all atoms, generally remaining below 0.6 nm. This indicates a stable structure with minimal deviations in atomic positions throughout the simulation, suggesting rigidity and stability. In contrast, the G320S variant showed higher fluctuations, particularly in specific regions where the RMSF exceeds 1 nm, indicating areas of significant flexibility within the protein structure. Similarly, the R223S variant also displayed increased fluctuations, with peaks surpassing 1 nm. Its fluctuation profile closely mirrors that of the G320S variant, indicating similar regions of flexibility (Figure 4)

#### 3.8.3. Radius of Gyration (Rg)

**Figure 5:**
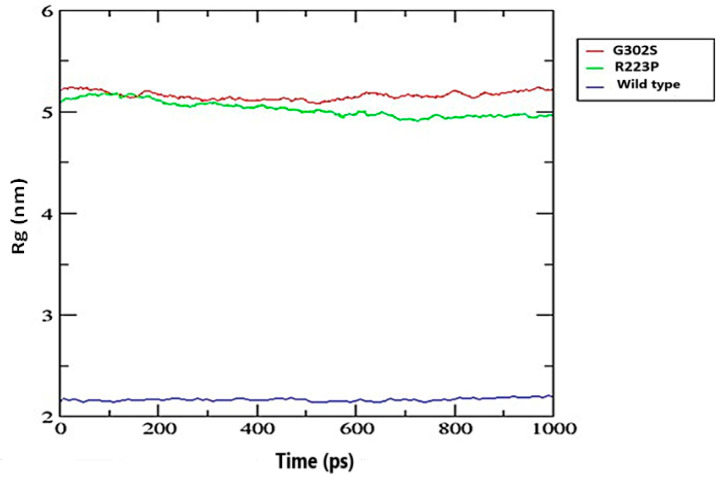
The Radius of Gyration (Rg) plot demonstrates the compactness of the wild-type protein compared to the G302S and R223P mutant variants. The Rg values, measured in nanometers (nm), are plotted on the y-axis, while the x-axis represents time in picoseconds (ps). The wild type is depicted in blue, with the G302S and R223P mutants shown in red and green, respectively.

The G320S variant’s radius of gyration fluctuated slightly above 5 nm, indicating moderate structural compactness and stability. Similarly, the R223’S variant’s Rg fluctuated around 5 nm, suggesting comparable stability to the G320S variant. In contrast, the wild-type variant has a significantly lower Rg, remaining steady at less than 2.5 nm throughout the observed period, indicating a much more compact and potentially more stable structure compared to the mutated variants (Figure 5).

#### 3.8.4. Number of Hydrogen Bonds over Time

**Figure 6:**
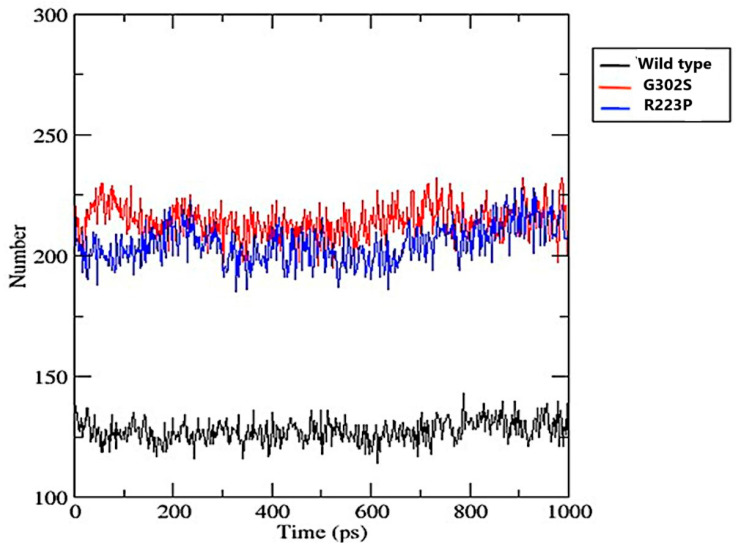
The number of hydrogen bonds over time for wild-type (black), G302S (red), and R223S (blue) protein variants. The y-axis represents the number of hydrogen bonds, while the x-axis represents the simulation time in picoseconds (ps).

The wild-type variant exhibited a significantly lower number of hydrogen bonds, averaging above 100 and below 150 throughout the duration. This suggests fewer interactions within the protein structure or with its environment compared to the mutated variants, which both showed a fluctuating but consistently higher number of hydrogen bonds generally ranging between about 200 and 250. This indicates more extensive intramolecular or intermolecular interactions (Figure 6).

#### 3.8.5. Solvent-Accessible Surface (SAS)

**Figure 7:**
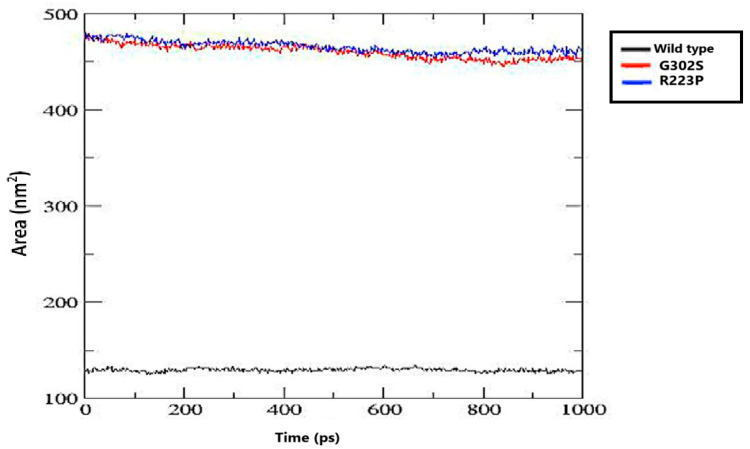
The solvent-accessible surface (SAS) of the protein over time for the wild type and two mutants (G302S and R223S). The SAS was calculated in nm^2^ over a simulation period of 1000 ps.

The wild-type protein shows a relatively low SAS value, stabilizing at around 120–130 nm^2^ throughout the simulation. Both G302S and R223S mutants exhibit significantly higher SAS values, averaging around 450–480 nm^2^. The difference suggests that both mutations induce changes in protein conformation that increase the exposed surface area, which could have implications for the protein’s stability or interactions with its environment (Figure 7).

### 3.9. 3D Simulation of EPO-R Protein Structural Changes

**Figure 8:**
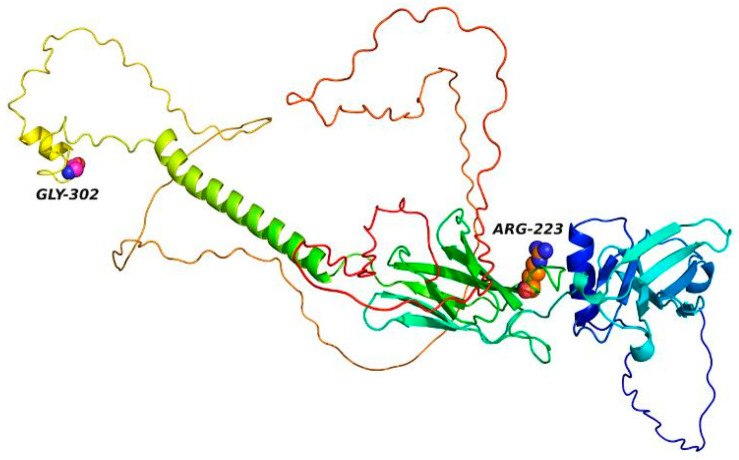
Three-dimensional structure of the EPO-R protein highlighting the two mutant residues, GLY-302 and ARG-223. The protein is color coded to represent different structural regions, with GLY-302 (magenta) and ARG-223 (orange) shown in ball-and-stick representations to indicate their positions within the protein structure.

The 3D structural changes of the EPO-R protein were displayed using PyMol 3.1 Software. Figure 8 shows the 3D structure of the wild type and two mutant variants, G320S and R223S, that are classified as deleterious using various bioinformatic tools and MDS.

## 4. Discussion

In this study, we conducted a comprehensive in silico analysis of missense nsSNPs in the *EPOR* gene to identify their structural and functional consequences on EPO-R protein. The study findings highlight the significant impact of certain nsSNPs on the EPO-R protein, providing insights into their potential pathogenicity.

Our analysis identified 34 out of 420 missense nsSNPs as deleterious using a suite of bioinformatics tools, including SIFT, PolyPhen-2, SNAP2, SNP & Go, PhD-SNP, and I-Mutant2.0. The significance of the identified deleterious nsSNPs is underscored by their potential effects on protein structure and stability [41]. This is especially relevant for EPO-R, where mutations can disrupt signaling pathways, potentially resulting in conditions like polycythemia vera and other myeloproliferative disorders [42].

Further analysis of the 34 SNPs using the MutPred tool revealed that 17 of them may disturb the molecular mechanisms; key mechanisms affected included gain of strand, gain of ADP-ribosylation, altered stability, altered transmembrane protein, loss of disulfide linkage, altered ordered interface, gain of O-linked glycosylation, loss of pyrrolidone carboxylic acid, gain of loop, altered disordered interface, gain of intrinsic disorder, loss of phosphorylation, and loss of sulfation. By detecting changes such as gain or loss of structural elements or post-translational modifications, MutPred assists in elucidating how these mutations can influence protein function at a molecular level. This is essential for uncovering the potential pathways by which nsSNPs contribute to the development of diseases [33].

The conservation of specific amino acid residues plays a crucial role in understanding protein stability, interactions, and overall function, providing essential views for the potential mechanisms through which nsSNPs contribute to disease pathogenesis [41]. Using the ConSurf tool to identify functionally conserved residues provides valuable insights into the structural and functional implications of nsSNPs [31]. In the present study, the analysis of nsSNPs by ConSurf revealed that two of the seventeen substitutions identified to disturb the molecular mechanisms (R223P and G302S) are located at functionally exposed conserved sites, indicating their critical roles in protein function. These substitutions can impact various aspects of protein biology and highlight the broad spectrum of functional consequences [42].

The result of MuPro tool reveals that the mutation R223P has a ΔΔG value of −1.62, indicating a significant decrease in stability. The substitution of arginine, a positively charged residue with proline, a rigid non-polar amino acid at position 223, likely disrupts local structural integrity and flexibility, which may affect the protein’s function. On the other hand, the mutation G302S has a ΔΔG value of −0.39, suggesting a moderate decrease in stability. Here, the substitution of glycine, a small flexible residue with serine that is a polar residue at position 302, introduces additional side-chain bulk and potential for hydrogen bonding, which could subtly disrupt the local structural conformation. However, the effect is less pronounced than R223P.

The analysis of protein properties using HOPE tool reveals that G302S substitution involves replacing a small, neutral glycine with a larger serine, which, despite being neutral in charge, disrupts local structural stability due to its size and inability to form the same hydrogen bonds. This substitution occurs near a highly conserved region, suggesting its critical role in protein function, with a MetaRNN score of 0.8904699 indicating a high probability of being deleterious. The R223P substitution changes a flexible, positively charged arginine to a smaller, hydrophobic proline, disrupting important ionic interactions and hydrogen bonds, including salt bridges with glutamic acids, and altering the protein’s stability. This mutation has a MetaRNN score of 0.8490628, also suggesting a substantial likelihood of a harmful impact. Radical amino acid changes, such as those seen in G302S and R223P, are more likely to face negative selection compared to conservative changes. This is because selective pressures often favor substitutions that retain similar properties, thereby maintaining the protein’s functional integrity [43]. The findings from our analysis align with previous studies that have shown that amino acid substitutions leading to significant alterations in charge or size can disrupt essential interactions within the protein, leading to misfolding, instability, or loss of function [44,45].

InterPro analysis of protein functional sites has highlighted key insights into the effects of R223P and G302S polymorphisms. The R223P variant resides within the fibronectin type III (FN3) domain, a structurally and functionally modular region marked by the Arg-Gly-Asp (RGD) motif essential for integrin binding, facilitating cell adhesion to the extracellular matrix [46,47,48]. This RGD sequence is pivotal for cell adhesion, migration, and signaling, which are essential for tissue development and repair [49,50]. Minor structural changes, such as the R223P mutation, could disrupt these functions, leading to altered cell responses and potential pathological outcomes [46,51].

The G302S polymorphism is located in the cytoplasmic domain of EPO-R, which is critical for interactions with signal transducers and protein tyrosine kinases. This domain’s role in signaling for erythropoiesis, or red blood cell formation, means that mutations here may impair receptor functionality and contribute to hematological disorders. The G302S variant could alter the receptor’s capacity to interact with downstream signaling molecules, affecting the erythropoietin-initiated signaling cascade [52,53].

The RMSD plot reveals significant structural deviations over time for the G302S and R223P variants compared to the wild type. While the wild type maintains low and stable RMSD values, indicating structural stability essential for ligand binding and receptor activation, the G302S and R223P variants show increased RMSD, suggesting structural instability. The G302S variant exhibits the highest RMSD, likely due to the replacement of glycine with serine, which introduces additional hydrogen bonding and disrupts local folding, potentially impairing ligand binding. Similarly, the R223P variant shows increased RMSD due to the rigid structure of the proline, which can cause kinks in the protein backbone, affecting EPO-R’s functional conformation. These structural instabilities in both variants could hinder EPO-R’s ability to bind erythropoietin or transmit signals effectively, suggesting that the mutations may lead to functional impairment in a biological context.

The radius of gyration (Rg) plot highlights differences in structural compactness and stability among the wild type and the two variants, G302S and R223P. The wild-type EPO-R maintains a consistently low Rg value, indicating a compact and stable structure essential for effective ligand binding and receptor activation. In contrast, the G302S variant shows a higher Rg value, suggesting a less compact structure that may disrupt the ligand-binding domain or overall receptor architecture, potentially impairing EPO-R’s ability to bind erythropoietin effectively. The R223P variant also exhibits a higher Rg than the wild type, though slightly lower than G302S, indicating some loss of compactness likely due to the rigidity of proline, which can induce structural kinks. These increased Rg values in both variants suggest reduced stability and compactness, which may compromise EPO-R’s structural integrity and functional role in erythropoiesis. [54].

Increased hydrogen bonds in G320S and R223P variants can be interpreted as an adaptive response to the structural perturbations caused by these mutations. For instance, the G320S mutation replaces glycine, a small and flexible residue, with serine, which introduces a polar side chain capable of forming additional hydrogen bonds. This change likely enhances local interactions within the protein, thereby compensating for any destabilizing effects associated with the mutation [55]. Moreover, the R223P mutation introduces proline, which is known to disrupt regular secondary structure due to its unique cyclic structure. This disruption can lead to increased flexibility in the protein backbone. However, the formation of additional hydrogen bonds in the vicinity of the R223P mutation may stabilize the overall structure by creating new interactions that counterbalance the flexibility introduced by proline [56]. Such findings are consistent with previous studies that have shown how mutations can lead to alterations in hydrogen bonding patterns, thereby affecting protein stability [57].

The wild-type protein maintains a relatively low SAS value, stabilizing at around 120–130 nm², which suggests a compact and stable conformation. In contrast, the G302S and R223S mutant variants show significantly higher SAS values, averaging between 450 and 480 nm². This substantial increase indicates that these mutations trigger conformational changes, exposing more of the protein’s surface area and potentially affecting its stability and interactions with other molecules in its environment [58].

The average minor allele frequency (MAF) of the mutant (T) allele of the SNP rs1321784132 (R223P) across all populations was quite low (0.000008), with the maximum observed frequency reaching 0.00004. Some populations, including the European, American, African, and Ashkenazi Jewish groups, exhibit a zero frequency for the mutant allele, indicating its rarity or even absence in these populations. In contrast, the Asian population shows a detectable frequency (0.00004), making it the group with the highest observed frequency for this mutation (https://www.ncbi.nlm.nih.gov/snp/rs1321784132) [accessed on 26 October 2024]. The rarity of the mutant allele (T) across most populations suggests that it could be associated with specific genetic backgrounds or environmental factors. The individuals carrying the allele in the Asian population might be at a higher risk for conditions related to *EPOR* gene function. Unfortunately, for the G allele of other SNP rs991881188 (G302S), the MAF was not available (https://www.ncbi.nlm.nih.gov/snp/?term=rs991881188) (accessed on 26 October 2024).

A limitation of this study was the in silico nature of the analysis. Further experimental research is recommended to fully understand the impact of these SNPs on EPO-R protein function and their association with related disorders.

## 5. Conclusions

Computational analysis of missense nsSNPs in the *EPOR* gene identified two variants, R223P and G302S, as deleterious. These variants are located in highly conserved regions and display ΔΔG values below zero, indicating reduced protein stability. Our results suggest that these SNPs could have significant effects on the structure and function of the EPO-R protein, potentially leading to pathogenic consequences.

## Figures and Tables

**Table 1:** Prediction of SNPs’ effect on protein structure and function using SIFT, PolyPhen-2, and SNAP2 tools.

Variant ID	Alleles	Amino Acid Change	SIFT		Polyphene2		SNAP2		
			Prediction	Score	Prediction	Score	Prediction	Score	Expected Accuracy
rs199645071	G>A	P380L	Deleterious	0.00	Probably	0.961	Effect	56	75%
rs750657898	A>G	L199P	Deleterious	0.05	Probably	1.000	Effect	19	56%
rs773564773	A>C	W233G	Deleterious	0.00	Probably	1.000	Effect	90	95%
rs1968317522	T>C	K301E	Deleterious	0.02	Probably	0.991	Effect	13	59%
rs139756642	G>A	P287L	Deleterious	0.00	Probably	1.000	Effect	50	75%
rs149831382	G>A	P168L	Deleterious	0.00	Probably	0.998	Effect	25	63%
rs192441411	A>C	L376R	Deleterious	0.00	Probably	1.000	Effect	59	75%
rs368363386	C>A	D351Y	Deleterious	0.00	Probably	1.000	Effect	3	53%
rs370541202	T>A	I464F	Deleterious	0.00	Probably	0.997	Effect	22	63%
rs373709817	C>T	V260M	Deleterious	0.02	Probably	0.999	Effect	37	66%
rs376951711	A>C	S465A	Deleterious	0.00	Probably	0.999	Effect	32	66%
rs533014098	A>G	L207P	Deleterious	0.00	Probably	1.000	Effect	71	85%
rs542643797	G>A	P239L	Deleterious	0.01	Probably	1.000	Effect	19	59%
	G>C	P239R	Deleterious	0.01	Probably	1.000	Effect	35	66%
rs751506215	G>A	R45W	Deleterious	0.00	Probably	0.987	Effect	39	66%
rs751621912	A>G	L93P	Deleterious	0.02	Probably	1.000	Effect	51	75%
rs752527298	T>A	D430V	Deleterious	0.00	Probably	1.000	Effect	53	75%
rs754199429	G>A	R100C	Deleterious	0.03	Probably	1.000	Effect	30	66%
rs757072422	C>T	E425K	Deleterious	00.0	Probably	1.000	Effect	23	63%
rs758272993	C>T	E336K	Deleterious	0.01	Probably	0.999	Effect	28	63%
rs760437132	A>C	L429R	Deleterious	0.00	Probably	0.999	Effect	54	75%
rs764303927	C>G	C52S	Deleterious	0.00	Probably	1.000	Effect	71	85%
rs765009836	C>T	E181K	Deleterious	0.01	Probably	1.000	Effect	66	80%
rs765615096	C>T	R202H	Deleterious	0.04	Probably	0.999	Effect	55	75%
rs771507239	C>A	D366Y	Deleterious	0.00	Probably	1.000	Effect	68	80%
rs771666923	C>T	V143M	Deleterious	0.01	Probably	1.000	Effect	28	63%
rs772238101	C>A	R165L	Deleterious	0.03	Probably	1.000	Effect	43	71%
rs775003412	T>C	Q305R	Deleterious	0.04	Probably	0.958	Effect	13	59%
rs776340905	G>A	N491K	Deleterious	0.00	Probably	1.000	Effect	17	59%
rs776800957	A>C	C52G	Deleterious	0.00	Probably	1.000	Effect	77	85%
rs779186064	A>G	W64R	Deleterious	0.00	Probably	1.000	Effect	91	95%
rs781454885	G>C	A40D	Deleterious	0.00	Probably	0.992	Effect	22	63%
rs781710022	A>T	I178N	Deleterious	0.00	Probably	1.000	Effect	61	80%
rs940691487	T>C	Y368C	Deleterious	0.00	Probably	1.000	Effect	53	75%
rs991881188	C>G	R223P	Deleterious	0.03	Probably	1.000	Effect	84	91%
rs1026783071	T>G	D467A	Deleterious	0.00	Probably	1.000	Effect	24	63%
rs1184535377	T>C	D372G	Deleterious	0.00	Probably	1.000	Effect	59	75%
rs1192368347	A>T	L257H	Deleterious	0.02	Probably	0.975	Effect	68	80%
rs1193366124	T>C	D461G	Deleterious	0.00	Probably	0.989	Effect	56	75%
rs1206022201	C>T	G471R	Deleterious	0.00	Probably	1.000	Effect	5	53%
rs1209147888	T>G	K453T	Deleterious	0.00	Probably	1.000	Effect	3	53%
rs1228428456	G>A	R179C	Deleterious	0.01	Probably	1.000	Effect	45	71%
rs1233264153	G>A	R215C	Deleterious	0.03	Probably	1.000	Effect	6	63%
rs1236502126	A>T	L266Q	Deleterious	0.00	Probably	1.000	Effect	28	63%
rs1254633566	C>A	V124F	Deleterious	0.01	Probably	0.997	Effect	69	80%
rs1277913272	G>A	S473F	Deleterious	0.00	Probably	0.998	Effect	25	63%
rs1281927241	G>A	P499S	Deleterious	0.00	Probably	0.999	Effect	14	59%
	G>T	P499T	Deleterious	0.00	Probably	0.999	Effect	19	59%
rs1291097518	C>G	E290Q	Deleterious	0.04	Probably	1.000	Effect	14	59%
rs1312478601	T>A	D351V	Deleterious	0.00	Probably	0.997	Effect	2	53%
rs1321784132	C>T	G302S	Deleterious	0.02	Probably	1.000	Effect	43	71%
rs1326443454	C>T	V182M	Deleterious	0.02	Probably	0.992	Effect	57	75%
rs1329852497	A>G	C107R	Deleterious	0.00	Probably	0.995	Effect	70	85%
rs1331043902	C>T	C107Y	Deleterious	0.00	Probably	1.000	Effect	77	85%
rs1335771561	A>G	C62R	Deleterious	0.00	Probably	0.994	Effect	78	85%
rs1368251390	A>G	L455P	Deleterious	0.00	Probably	0.995	Effect	27	63%
rs1393553623	A>C	Y216D	Deleterious	0.00	Probably	1.000	Effect	91	95%
rs1404996393	T>C	Y504C	Deleterious	0.00	Probably	1.000	Effect	46	71%
rs1436380909	A>G	V260A	Deleterious	0.05	Probably	0.976	Effect	8	53%
rs1453095403	G>A	R221C	Deleterious	0.00	Probably	1.000	Effect	85	91%
rs1465679458	T>G	E402D	Deleterious	0.00	Probably	0.976	Effect	2	53%
rs1471802731	G>T	P484H	Deleterious	0.00	Probably	0.996	Effect	6	53%
rs1568328293	C>A	V333F	Deleterious	0.01	Probably	1.000	Effect	66	80%
rs1968305256	T>C	Y489C	Deleterious	0.00	Probably	1.000	Effect	34	66%
rs1968305306	A>T	Y489N	Deleterious	0.00	Probably	1.000	Effect	67	80%
rs1968306772	C>T	G471E	Deleterious	0.00	Probably	1.000	Effect	30	66%
rs1968306993	T>C	Y468C	Deleterious	0.00	Probably	1.000	Effect	54	75%
rs1968307255	A>G	I464T	Deleterious	0.00	Probably	0.997	Effect	24	63%
rs1968310587	T>A	D398V	Deleterious	0.00	Probably	0.997	Effect	32	66%
	T>C	D398G	Deleterious	0.00	Probably	0.988	Effect	15	59%
rs1968314573	C>G	E332Q	Deleterious	0.02	Probably	1.000	Effect	50	75%
rs1968315618	A>G	C314R	Deleterious	0.05	Probably	0.996	Effect	5	53%
rs1968317423	T>A	N303Y	Deleterious	0.00	Probably	1.000	Effect	41	71%
rs1968318332	G>A	P284L	Deleterious	0.00	Probably	1.000	Effect	55	75%
rs1968345368	G>A	R275C	Deleterious	0.01	Probably	1.000	Effect	59	75%
rs1968350306	G>A	R223C	Deleterious	0.00	Probably	1.000	Effect	60	80%
rs1968351370	T>A	N209I	Deleterious	0.05	Probably	0.988	Effect	51	75%
rs1968351718	T>C	T203A	Deleterious	0.04	Probably	0.967	Effect	38	66%
rs1968363098	T>C	E181G	Deleterious	0.03	Probably	1.000	Effect	70	85%
rs1968364624	C>A	G160V	Deleterious	0.05	Probably	0.974	Effect	5	53%

**Table 2:** Prediction of the SNPs-disease association using SNPs & Go and PhD-SNP tools.

Accession No.	Substitution	Amino Acid Change	SNPs & Go		PhD-SNP	
			Prediction	R1	Prediction	Score
rs750657898	A>G	L199P	Disease	7	Disease	5
rs773564773	A>C	W233G	Disease	6	Disease	2
rs192441411	A>C	L376R	Disease	7	Disease	4
	A>T	L376Q	Disease	6	Disease	4
rs368363386	C>A	D351Y	Disease	4	Disease	1
rs533014098	A>G	L207P	Disease	8	Disease	6
rs542643797	G>A	P239L	Disease	5	Disease	0
rs751506215	G>A	R45W	Disease	1	Disease	3
	G>C	R45G	Disease	0	Disease	1
rs751621912	A>G	L93P	Disease	8	Disease	1
rs752527298	T>A	D430V	Disease	6	Disease	1
rs754199429	G>A	R100C	Disease	7	Disease	5
rs760437132	A>C	L429R	Disease	8	Disease	4
rs764303927	C>G	C52S	Disease	9	Disease	3
rs765009836	C>T	E181K	Disease	6	Disease	3
rs765615096	C>T	R202H	Disease	5	Disease	3
rs771507239	C>A	D366Y	Disease	8	Disease	4
rs776800957	A>C	C52G	Disease	9	Disease	5
rs779186064	A>G	W64R	Disease	9	Disease	1
rs781710022	A>T	I178N	Disease	6	Disease	3
rs940691487	T>C	Y368C	Disease	8	Disease	5
rs991881188	C>G	R223P	Disease	8	Disease	6
rs1184535377	T>C	D372G	Disease	4	Disease	2
rs1192368347	A>T	L257H	Disease	1	Disease	1
rs1193366124	T>C	D461G	Disease	2	Disease	1
rs1228428456	G>A	R179C	Disease	7	Disease	3
rs1233264153	G>A	R215C	Disease	8	Disease	5
rs1236502126	A>T	L266Q	Disease	5	Disease	3
rs1254633566	C>A	V124F	Disease	4	Disease	3
rs1312478601	T>A	D351V	Disease	2	Disease	1
rs1321784132	C>T	G302S	Disease	6	Disease	2
rs1329852497	A>G	C107R	Disease	9	Disease	4
rs1331043902	C>T	C107Y	Disease	7	Disease	4
rs1335771561	A>G	C62R	Disease	9	Disease	6
rs1368251390	A>G	L455P	Disease	7	Disease	3
rs1393553623	A>C	Y216D	Disease	8	Disease	3
rs1453095403	G>A	R221C	Disease	8	Disease	4
rs1471802731	G>T	P484H	Disease	3	Disease	0
rs1968305256	T>C	Y489C	Disease	5	Disease	2
rs1968305306	A>T	Y489N	Disease	5	Disease	0
rs1968306772	C>T	G471E	Disease	4	Disease	2
rs1968306993	T>C	Y468C	Disease	4	Disease	2
rs1968310587	T>A	D398V	Disease	4	Disease	2
rs1968315618	A>G	C314R	Disease	9	Disease	6
rs1968317423	T>A	N303Y	Disease	6	Disease	2
rs1968345368	G>A	R275C	Disease	6	Disease	4
rs1968350306	G>A	R223C	Disease	7	Disease	5
rs1968351370	T>A	N209I	Disease	6	Disease	3
rs1968364624	C>A	G160V	Disease	2	Disease	4

**Table 3:** Prediction of SNPs effect on protein stability using the I-Mutant and MuPro tools.

Accession No.	Substitution	Amino Acid Change	I mutant		MuPro	
			Prediction	R1	Prediction	ΔΔG
rs750657898	A>G	L199P	Decrease	5	Decrease stability	−1.583
rs773564773	A>C	W233G	Decrease	9	Decrease stability	−0.995
rs192441411	A>C	L376R	Decrease	2	Decrease stability	−1.345
	A>T	L376Q	Decrease	7	Decrease stability	−1.30
rs533014098	A>G	L207P	Decrease	4	Decrease stability	−1.57
rs542643797	G>A	P239L	Decrease	5	Decrease stability	−0.04
	G>C	P239R	Decrease	6	Decrease stability	−0.69
rs751506215	G>A	R45W	Decrease	4	Decrease stability	−1.20
	G>C	R45G	Decrease	8	Decrease stability	−1.73
rs751621912	A>G	L93P	Decrease	6	Decrease stability	−2.22
rs752527298	T>A	D430V	Decrease	0	Decrease stability	−0.20
rs754199429	G>A	R100C	Decrease	5	Decrease stability	−0.47
rs760437132	A>C	L429R	Decrease	7	Decrease stability	−1.75
rs764303927	C>G	C52S	Decrease	7	Decrease stability	−1.92
rs765009836	C>T	E181K	Decrease	7	Decrease stability	−1.35
rs765615096	C>T	R202H	Decrease	7	Decrease stability	−0.89
rs776800957	A>C	C52G	Decrease	8	Decrease stability	−2.16
rs779186064	A>G	W64R	Decrease	7	Decrease stability	−0.60
rs781710022	A>T	I178N	Decrease	4	Decrease stability	−1.56
rs940691487	T>C	Y368C	Decrease	1	Decrease stability	−1.26
rs991881188	C>A	R223L	Decrease	3	Decrease stability	−0.49
	C>G	R223P	Decrease	3	Decrease stability	−1.62
rs1184535377	T>C	D372G	Decrease	3	Decrease stability	−1.80
rs1192368347	A>T	L257H	Decrease	3	Decrease stability	−2.04
rs1193366124	T>C	D461G	Decrease	8	Decrease stability	−1.27
rs1233264153	G>A	R215C	Decrease	5	Decrease stability	−0.48
rs1236502126	A>T	L266Q	Decrease	4	Decrease stability	−1.91
rs1254633566	C>A	V124F	Decrease	9	Decrease stability	−0.75
rs1321784132	C>T	G302S	Decrease	5	Decrease stability	−0.39
rs1329852497	A>G	C107R	Decrease	2	Decrease stability	−0.97
rs1331043902	C>T	C107Y	Decrease	1	Decrease stability	−0.76
rs1368251390	A>G	L455P	Decrease	1	Decrease stability	−2.20
rs1393553623	A>C	Y216D	Decrease	3	Decrease stability	−1.17
rs1453095403	G>A	R221C	Decrease	1	Decrease stability	−0.01
rs1471802731	G>T	P484H	Decrease	8	Decrease stability	−1.16
rs1968305256	T>C	Y489C	Decrease	0	Decrease stability	−0.89
rs1968305306	A>T	Y489N	Decrease	8	Decrease stability	−1.20
rs1968364624	C>A	G160V	Decrease	4	Decrease stability	−0.60

**Table 4:** Prediction of the molecular mechanisms of pathogenicity using the MutPred tool.

Accession No.	Substitution	Amino Acid Change	MutPred		*p* Value
			Score	Molecular Mechanism with *p*-Values ≤ 0.05	
rs750657898	A>G	L199P	0.606	Gain of strand	0.02
				Gain of ADP-ribosylation at R202	0.04
				Altered stability	0.04
rs533014098	A>G	L207P	0.864	Gain of strand	0.02
rs542643797	G>A	P239L	0.501	Altered transmembrane protein	0.04
	G>C	P239R	0.558	Altered transmembrane protein	0.02
rs751621912	A>G	L93P	0.825	Loss of disulfide linkage at C91	0.04
				Gain of strand	0.03
				Altered stability	0.01
rs764303927	C>G	C52S	0.929	Altered ordered interface	0.02
rs779186064	A>G	W64R	0.955	Loss of strand	0.03
				Loss of disulfide linkage at C62	0.01
rs781710022	A>T	I178N	0.639	Loss of strand	0.04
rs991881188	C>G	R223P	0.841	Loss of strand	0.04
rs1192368347	A>T	L257H	0.713	Altered transmembrane protein	0.03
rs1193366124	T>C	D461G	0.732	Gain of O-linked glycosylation at S462	0.03
rs1321784132	C>T	G302S	0.672	Loss of pyrrolidone carboxylic acid at Q305	0.05
				Gain of loop	0.04
rs1329852497	A>G	C107R	0.902	Loss of disulfide linkage at C107	0.01
				Altered disordered interface	0.04
				Altered transmembrane protein	0.04
rs1331043902	C>T	C107Y	0.870	Loss of disulfide linkage at C107	0.01
				Gain of loop	0.04
				Altered transmembrane protein	0.03
rs1368251390	A>G	L455P	0.624	Gain of intrinsic disorder	0.02
				Altered disordered interface	0.04
rs1393553623	A>C	Y216D		Altered stability	0.03
rs1968305256	T>C	Y489C	0.522	Loss of phosphorylation at Y485	0.02
				Loss of sulfation at Y489	0.02
rs1968305306	A>T	Y489N	0.696	Loss of phosphorylation at Y485	0.03
				Loss of sulfation at Y489	0.02

## Data Availability

Data concerning missense nsSNPs in the EPOR gene are available at the dbSNP database (http://www.ncbi.nlm.nih.gov/SNP/) (accessed on 11 November 2023). The protein sequence for EPO-R (P19235) is available at the UniProt database (http://www.uniprot.org/uniprot/) (accessed on 11 November 2023).
